# Optimization of Mixed Inulin, Fructooligosaccharides, and Galactooligosaccharides as Prebiotics for Stimulation of Probiotics Growth and Function

**DOI:** 10.3390/foods12081591

**Published:** 2023-04-09

**Authors:** Ekkachai Kaewarsar, Chaiyavat Chaiyasut, Narissara Lailerd, Netnapa Makhamrueang, Sartjin Peerajan, Sasithorn Sirilun

**Affiliations:** 1 Department of Pharmaceutical Sciences, Faculty of Pharmacy, Chiang Mai University, Chiang Mai 50200, Thailand; ekkachai_kaew@cmu.ac.th (E.K.); chaiyavat@gmail.com (C.C.); netnapa.ma@cmu.ac.th (N.M.); 2 Innovation Center for Holistic Health, Nutraceuticals and Cosmeceuticals, Faculty of Pharmacy, Chiang Mai University, Chiang Mai 50200, Thailand; 3 Department of Physiology, Faculty of Medicine, Chiang Mai University, Chiang Mai 50200, Thailand; narissara.lailerd@cmu.ac.th; 4 Health Innovation Institute, Chiang Mai 50200, Thailand; s.peerajan@gmail.com

**Keywords:** colonic food, gut microbiota, oligosaccharides, prebiotics, probiotics, synbiotics

## Abstract

Prebiotics have become an important functional food because of their potential for modulating the gut microbiota and metabolic activities. However, different prebiotics can stimulate the growth of different probiotics. The optimization of prebiotics was focused on in this study in order to stimulate the representative probiotics’ growth (*Lacticaseibacillus rhamnosus* (previously *Lactobacillus rhamnosus*) and *Bifidobacterium animalis* subsp. *lactis*) and their function. The culture medium was supplemented with three prebiotics, including inulin (INU), fructooligosaccharides (FOS), and galactooligosaccharides (GOS). All prebiotics can clearly stimulate the growth of probiotic strains in both monoculture and co-culture. The specific growth rates of *L. rhamnosus* and *B. animalis* subsp. *lactis* were shown in GOS (0.019 h^−1^) and FOS (0.023 h^−1^), respectively. The prebiotic index (PI) scores of INU (1.03), FOS (0.86), and GOS (0.84) in co-culture at 48 h were significantly higher than the control (glucose). The mixture of prebiotics to achieve high quality was optimized using the Box–Behnken design. The optimum prebiotic ratios of INU, FOS, and GOS were 1.33, 2.00, and 2.67% *w*/*v*, respectively, with the highest stimulated growth of probiotic strains occurring with the highest PI score (1.03) and total short chain fatty acid concentration (85.55 µmol/mL). The suitable ratio of mixed prebiotics will function as a potential ingredient for functional foods or colonic foods.

## 1. Introduction

The microbiota in the gut tract is composed of trillions of microorganisms, including bacteria, viruses, and fungi, playing a crucial role in the digestive system functioning and overall health [1]. A healthy balance of microorganisms can help to prevent the overgrowth of harmful microorganisms, leading to infection and inflammation. An imbalance in the microbiota has been linked to a range of health problems, including autoimmune disorders, cardiovascular disease, certain types of cancer, obesity, and other metabolic disorders [2,3,4]. Presently, the study of the balance of gut microbiota relies on cutting-edge techniques, allowing researchers to better understand the composition, function, interactions of the gut microbiota with the host, and techniques for a specific person by the precision microbiota [5].

Prebiotics are defined as “a substrate that is selectively utilized by host microorganisms conferring a health benefit” [6]. Prebiotics are not hydrolyzed or absorbed in the upper part of the gastrointestinal tract (stomach and small intestine), and they should be selectively absorbed by the limited beneficial microorganisms when they reach the large intestine [7,8,9] Furthermore, prebiotics are metabolized by the gut microbiota, which can ferment and digest prebiotics by the beta-glucosidase enzyme into short chain fatty acids (SCFAs). They consist of acetate, propionate, and butyrate, which are used by the host as an energy source [8]. The SCFAs are absorbed by epithelial cells for use as an energy source and as metabolic regulators, improving villi growth, crypt development, tight junctions, and mucin production [10]. For example, butyrate affects the formation of the intestinal epithelium [11]. Through the control of nutrient and ion transporters, SCFAs are also important for promoting salt and water absorption in the colon, which can also prevent diarrhea caused by short-bowel syndrome (SBS) [12]. SCFAs influence the drop in pH of the gut, inhibiting the growth of microbial pathogens [9,10]. The gut microbiota, or beneficial bacteria in the intestine, can produce antimicrobial compounds to inhibit pathogenic bacteria and degrade prebiotics into oligosaccharides or monosaccharides that can attach with the lectin receptor on the epithelial cell surface resulting in block pathogenic bacteria colonization at the receptor site [10]. Moreover, some gut microbiota can produce antimicrobial factors or stimulate the immune system by signaling dendritic cells [9,10].

The consumption of prebiotics affects gut microbiota composition and metabolic activity. The molecular structure of prebiotics determines their physiological effect and the types of gut microbiota that can use prebiotics as carbon sources and energy in the intestines [9]. Moreover, prebiotic properties are associated with changes in the gut microbiota and improvements in metabolic parameters related to obesity, inflammation, glucose homeostasis disorders, and abnormal plasma lipid levels [13]. Additionally, prebiotics indirectly cause a decrease in triglyceride levels in the serum and may affect mineral absorption in the large intestine, protecting against inflammatory bowel syndrome by stimulating butyrate production [14]. Therefore, prebiotics have become an important functional food called “colonic food”, which can improve health through the colonic microbiota [15]. As the stimulation of well-known probiotic strains leads to the selection of the most profitable combination between substrate and microorganism, the ingestion of suitably chosen probiotics and prebiotics may boost the positive effects, both individually and synergistically [16]. Most prebiotics may be used without danger; however, when consumed in excess, they might induce negative effects such as bloating, gas, and gastrointestinal discomfort [11].

Prebiotics are available in a variety of forms, with the most commonly used among humans being inulin (INU), fructooligosaccharides (FOS), and galactooligosaccharides (GOS) [9,17]. Moreover, they are widely accepted as prebiotics, a fact supported in many human trials [18,19]. One related study reported that FOS and GOS were tested in vivo for all the requirements of the current criteria for successful prebiotics [20]. In addition, related studies revealed that at least 4 g/day but preferably 8 g/day of FOS would be required to significantly raise bifidobacteria in the human intestines [21]. Thus, mixing prebiotics in an optimal ratio may increase development, specifically stimulating the growth of probiotics and their activity. Moreover, the use of prebiotics alone or mixing prebiotics and probiotics, by selectively stimulating the growth and/or activating the metabolism of one or a limited number of health-promoting bacteria, can improve host welfare. These synbiotics are recognized to have the ability to promote and improve the gastrointestinal health of humans [15].

Therefore, the purpose of this study was to optimize the composition of a mixture of prebiotics for probiotic growth stimulation and combine it with probiotics for functional use as a human symbiotic formulation. The experiments were designed in a ratio of three prebiotics, including INU, FOS, and GOS. The growth of representative probiotic strains (*Lactobacillus* or *Lacticaseibacillus* and *Bifidobacterium*), the prebiotic index (PI), and the total SCFA concentration, including acetic acid, propionic acid, and butyric acid, were used as response effects of the prebiotic mixtures to select an optimal prebiotic ratio.

## 2. Materials and Methods

### 2.1. Bacterial Strains

Representative probiotics, *Lacticaseibacillus rhamnosus* (previously *Lactobacillus rhamnosus* [22]) strain HII117 (100% similarity with accession number as gi: NR_113332.1) and *Bifidobacterium animalis* subsp. *lactis*, were obtained from the Innovation Center for Holistic Health, Nutraceuticals, and Cosmeceuticals, Faculty of Pharmacy, Chiang Mai University. *L. rhamnosus* were grown anaerobically at 37 °C for 24–48 h after being cultured in De Man, Rogosa, and Sharpe (MRS) broth (10% *v*/*v*), while *Bifidobacterium animalis* subsp. *lactis* was cultured in MRS supplemented with L-cysteine hydrochloride monohydrate (HiMedia Laboratories, Mumbai, India). The pathogenic bacteria, including *Escherichia coli* ATCC 25922, and *S. entericaa* subsp. *enterica* ser. Typhi DMST 22842, were obtained from the culture collection of the Faculty of Pharmacy, Chiang Mai University (CMU), Thailand. All pathogenic bacteria were cultured (10% *v*/*v*) in Brain Heart Infusion (BHI) medium (HiMedia Laboratories, Mumbai, India) and incubated at 37 °C for 24 h.

After that, the bacterial strains were centrifuged for 5 min at a speed of 9950× *g* at a temperature of 4 °C. The bacterial pellets were rinsed twice with a pH 6.8 phosphate buffer solution (PBS) by centrifugation at 9950× *g*, 4 °C for 5 min. The strains were maintained in a suitable culture medium with 20% glycerol before being kept at −20 °C until the experiment.

### 2.2. Preparation of Prebiotics

The three types of commercial oligosaccharides consist of INU (BENEO-Orafti, Oreye, Belgium), FOS (BENEO-Orafti, Oreye, Belgium), and GOS (New Francisco Biotechonology Co., Ltd., Yunfu, China). A 2% *w/v* stock solution of prebiotics was prepared in deionized water and filtered through a 0.22 µm syringe filter (CNW technologies, Shanghai, China) [23].

### 2.3. Determination of the Growth Rate and the Specific Growth Rate

A sterilized MRS culture medium (10 g/L peptone, 8 g/L beef extract, 4 g/L yeast extract, 2 g/L ammonium citrate, 1 g/L polysorbate 80, 5 g/L sodium acetate, 0.1 g/L magnesium sulfate heptahydrate, 0.05 g/L manganese sulfate monohydrate, and 2 g/L potassium hydrogen phosphate) was supplemented with the prebiotic solutions (INU, FOS, and GOS) as a carbon source, which are denoted by the letters M-INU, M-FOS, and M-GOS, respectively. A positive control experiment was carried out using a culture medium that contained glucose (M-GLU), while MRS media without glucose and prebiotic solution were used as a negative control (M-MRS). The 10^6^ CFU/mL of bacterial strains and co-culturing strains were then cultured in the different medium and incubated at 37 °C for 48 h. The plate count colony technique was used to determine the growth rate at 0, 6, 12, 18, 24, 36, and 48 h.

The specific growth rate period was referred to as the rise rate of biomass of a cell population per unit in biomass concentration. The bacterial cultures, *L. rhamnosus* and *B. animalis* subsp. *lactis*, were centrifuged at 9950× *g* for 5 min at 4 °C before being rinsed twice with PBS. The pellets were diluted by a 10-fold PBS buffer dilution. The dilution of each probiotic, 1 mL, was poured onto its own culture medium plate, which was then placed in an incubator at 37 °C for 24–48 h. The specific growth rate (µ) was calculated using the following Equation (1) [23]:

µ = (ln x_1_ − ln x_2_)/(t_1_ − t_2_), (1)

where t_1_ and t_2_ were the log phase period of the bacteria growth, x_1_ was the number of bacteria at time t_1_, and x_2_ was the number of bacteria at time t_2_.

### 2.4. Determination of Organic Acid

The concentrations of lactic acid, acetic acid, propionic acid, and butyric acid were determined using high-performance liquid chromatography (HPLC) [20,24,25,26]. The bacterial cultures and co-cultures were prepared by centrifuging at 9950× *g* for 5 min at 4 °C. The supernatant (500 µL) was thoroughly combined with 500 µL of 5 mM sulfuric acid (RCI Labscan, Bangkok, Thailand) in the tube. The solution was filtered through a 0.22-µm filter (CNW technologies, Shanghai, China) and kept in an amber glass vial tube for analysis. These organic acids were identified using a SUGAR column (6 µm, 8 × 300 mm, SH1011, Shodex, Munich, Germany) with an HPLC system (model LC-20AD, Shimadzu, Kyoto, Japan). The analytical column was placed at a constant temperature of 75 °C. The mobile phase, 5 mM sulfuric acid, was passed through a filter (CNW technologies, Shanghai, China) and degassed for 30 min in an ultrasonic bath (Trassonic Digital S, Elma, Singen, Germany) before the operation. The flow rate of the mobile phase was 0.6 mL/min in the gradient program. The organic acids were detected by an ultraviolet detector at 220 nm. The standard substances of lactic acid, acetic acid, propionic acid, and butyric acid were obtained from LOBA Chemie (Mumbai, India), RCI Labscan (Bangkok, Thailand), Ajax Finechem Pty (Seven Hills, New South Wales, Australia), and PanReac AppliChem (Darmstadt, South Hesse, Germany).

### 2.5. Determination of Prebiotic Index (PI)

The prebiotic index was determined by co-culture between probiotics (*L. rhamnosus* and *B. animalis* subsp. *lactis*) and pathogenic bacteria (*E. coli* and *S.* Typhi) (10^6^ CFU/mL) in an MRS medium supplemented with glucose (M-GLU) and prebiotics (M-INU, M-FOS, and M-GOS). The co-culture was anaerobically incubated at 37 °C for 48 h. The colonies were then cultured on selective media: MRS agar supplemented with bromocresol purple (Fisher Scientific, Loughborough, UK) under anaerobic conditions at 37 °C for *L. rhamnosus* and Bifidus selective media (BSM) agar (Fluka, Sigma-Aldrich, St. Louis, MO, USA) under strictly anaerobic conditions at 37 °C for *B. animalis* supsp. *Lactis*. The bacterial colonies were counted and the index score was calculated according to the following Equation (2):

PI = (Lac + Bif − Eco − Sal)/Total,(2)

where PI value was calculated by comparing the increase in the growth of representative probiotic bacteria (Lac and Bif) to the growth of representative gut bacterial pathogens (Eco and Sal) in the presence of oligosaccharides. Lac is the log number (CFU/mL) of *L. rhamnosus* at sampling times divided by log number (CFU/mL) at baseline (time 0), Bif is the log number (CFU/g) of *B. animalis* subsp. *lactis* at sampling times divided by log number (CFU/mL) at baseline (time 0), Eco is log number (CFU/mL) of *E. coli* at sampling times divided by log number (CFU/g) at baseline (time 0), Sal is log number (CFU/mL) of *S.* Typhi at sampling times divided by log number (CFU/mL) at baseline (time 0), and Total is log number (CFU/mL) of total bacteria at sampling times divided by log number (CFU/mL) at baseline (time 0) [27].

### 2.6. Optimization of Prebiotics Ratio by the Experimental Design

The ratio percentage of prebiotics was varied in MRS medium as a carbon source. Experiments were performed with three variables, including INU (X_1_), FOS (X_2_), and GOS (X_3_). The variables with code levels of −1, 0, and 1 had low, medium, and high prebiotic content, respectively. The range of prebiotic content was 1.33–2.67% *w/v* [21]. A Box–Behnken design (BBD) was utilized for the optimization of the prebiotic ratio using Design Expert software (version 10, Stat-Ease Inc., Minneapolis, MN, USA), leading to a total of 17 runs (Table 1). An analysis of variance (ANOVA) was used to obtain the results, which were based on the *p*-value at the 95% confidence level. PI score and total short chain fatty acids (SCFAs) (acetic acid, propionic acid, and butyric acids) were assessed as responses to the experimental design.

### 2.7. Fourier Transform Infrared Spectroscopy (FTIR) Analysis

The phytochemical structure of the optimal prebiotic ratio was analyzed by using FTIR with an IR microscope (NICOLET 6700 FT-IR, Thermo Science Waltham, MA, USA) at a spectra range of 4000 to 400 cm^−1^ using KBr pellets, and the resolution was 2 cm^−1^ [28,29,30].

### 2.8. Statistical Analysis

All results of each experiment were determined in triplicate and expressed as mean values with standard deviations (SD). ANOVA and post hoc Turkey HSD multiple comparisons among means were performed using statistical SPSS software (version 17, SPSS Inc., Chicago, IL, USA) to analyze the significant differences in the growth of probiotics, PI scores, and organic acids between the different prebiotics. *p <* 0.05 was considered statistically significant.

## 3. Results

### 3.1. Kinetics of Monoculture Bacterial Growth on Different Media Supplemented Prebiotics

**Figure 1:**
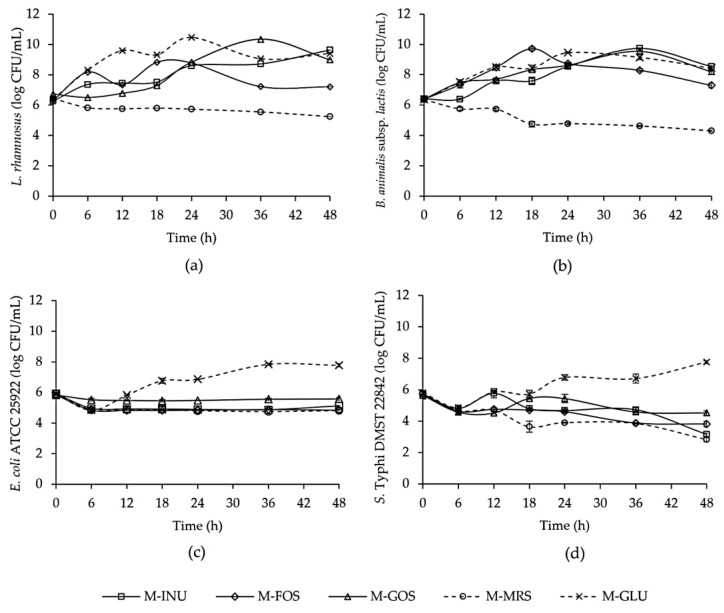
The growth kinetics of probiotics and pathogenic bacteria, (**a**) *L. rhamnosus*, (**b**) *B. animalis* subsp. *lactis*, (**c**) *E. coli* ATCC 25922, and (**d**) *S*. Typhi DMST 22842 in MRS medium and the different mediums supplemented with glucose (control) and prebiotics.

Plate dilution counts were used to determine growth parameters, which were expressed as log CFU/mL. As shown in Figure 1, the trend of growth kinetics had decreased for all bacteria in M-MRS, whereas the trend had increased for all bacteria in M-GLU. At the start of the experiment (0 h), the growth kinetics of *L. rhamnosus, B. animalis* subsp. *lactis*, *E. coli*, and *S.* Typhi in culture media without carbon source and prebiotics (M-MRS) were, consecutively, 6.43 ± 0.02, 6.36 ± 0.04, 5.92 ± 0.04, and 5.73 ± 0.04 log CFU/mL. After incubation in M-MRS for 48 h, *L. rhamnosus, B. animalis* subsp. *lactis, E. coli*, and *S.* Typhi were decreased to 5.24 ± 0.02, 4.30 ± 0.04, 4.82 ± 0.11, and 2.82 ± 0.12 log CFU/mL, respectively. Considering probiotic bacteria culturing in M-GLU, the growth of *L. rhamnosus* and *B. animalis* subsp. *lactis* was 6.40 ± 0.06 and 6.37 ± 0.07 log CFU/mL, respectively, at the beginning of incubation. Following that, greater levels of growth were observed 24 h after incubation: 10.46 ± 0.05 log CFU/mL for *L. rhamnosus* and 9.45 ± 0.02 log CFU/mL for *B. animalis* subsp. *lactis*. For pathogen bacteria, *E. coli* and *S.* Typhi were cultured in M-GLU at a range of 4.84 ± 0.04 to 7.78 ± 0.10 and 4.85 ± 0.16 to 7.76 ± 0.09 log CFU/mL, respectively, of which the lowest level was at 6 h and the highest level was at 48 h.

The culture medium containing various prebiotics (Figure 1a,b), including M-INU, M-FOS, and M-GOS, clearly stimulated the growth of probiotic strains. The growth number of *L. rhamnosus* in M-INU continuously increased from 6.26 ± 0.02 to 9.62 ± 0.02 log CFU/mL during 48 h of incubation. The counts of *L. rhamnosus* ranged from 6.31 ± 0.02 (0 h) to 8.82 ± 0.03 log CFU/mL (18 h) in M-FOS and 6.73 ± 0.05 (0 h) to 10.34 ± 0.04 CFU/mL (36 h) in M-GOS. After the initial culture period, *B. animalis* subsp. *lactis* in M-INU and M-GOS reached maximums of 9.74 ± 0.04 and 9.54 ± 0.15 log CFU/mL, respectively, at 36 h of incubation time. However, *B. animalis* subsp. *lactis* in M-FOS exhibited the highest number at 18 h of incubation (9.72 ± 0.11 log CFU/mL) and the lowest number at 0 h of incubation (6.39 ± 0.05 log CFU/mL).

As a result of *E. coli* (Figure 1c) and *S.* Typhi (Figure 1d), the growth trend of culture medium containing different prebiotics decreased over the incubation period. The number of *E. coli* at the beginning of the culture (0 h) was 5.92 ± 0.03 log CFU/mL in M-INU, 5.83 ± 0.14 log CFU/mL in M-FOS, and 5.84 ± 0.02 log CFU/mL in M-GOS. After being cultured with M-INU, M-FOS, and M-GOS for 48 h of incubation time, the growth of *E. coli* decreased to 5.12 ± 0.04, 4.84 ± 0.14, and 5.58 ± 0.10 log CFU/mL, respectively. However, the minimal numbers of *E. coli* found in M-INU, M-FOS, and M-GOS were as follows: 4.86 ± 0.02 log CFU/mL (at 36 h), 4.82 ± 0.06 log CFU/mL (at 18 h), and 5.45 ± 0.09 log CFU/mL (at 18 h), respectively. The numbers of *S.* Typhi at 0 h of incubation time were 5.72 ± 0.24 log CFU/mL in M-INU, 5.75 ± 0.10 log CFU/mL in M-FOS, and 5.64 ± 0.24 log CFU/mL in M-GOS. The growth numbers in M-INU, M-FOS, and M-GOS continuously decreased to 3.14 ± 0.09, 3.81 ± 0.19, and 4.52 ± 0.14 log CFU/mL, respectively, at the end of the experiment.

### 3.2. Specific Growth Rate of Monoculture Probiotics on Different Medium Supplemented Prebiotics

**Figure 2:**
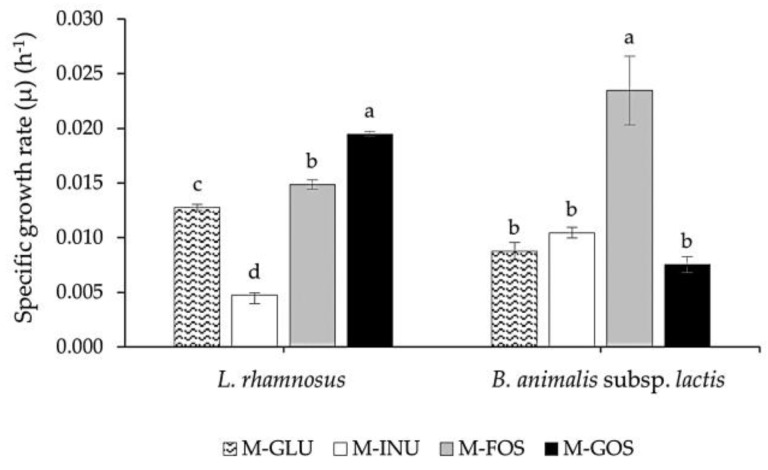
The specific growth rates of *L. rhamnosus* and *B. animalis* subsp. *lactis* in the different mediums supplemented with glucose (control) and prebiotics, for which the various letters showed a significant mean difference (*p* < 0.05) in each probiotic strain.

Figure 2 displays that *L. rhamnosus* in M-GOS *(*µ *=* 0.019 h^−1^*)* resulted in the highest value that was significantly different (*p* < 0.05), followed by M-FOS (µ = 0.015 h^−1^), M-GLU (µ = 0.013 h^−1^), and M-INU (µ = 0.005 h^−1^). The specific growth rate of *B. animalis* subsp. *lactis* in M-FOS (µ = 0.023 h^−1^) showed the most significant difference (*p* < 0.05) while the specific growth rates in M-GLU, M-INU, and M-GOS were 0.009, 0.010, and 0.008 h^−1^, respectively, without significant differences (*p* > 0.05).

### 3.3. Kinetics of Co-Culture Bacterial Growth on Different Medium Supplemented Prebiotics

**Figure 3:**
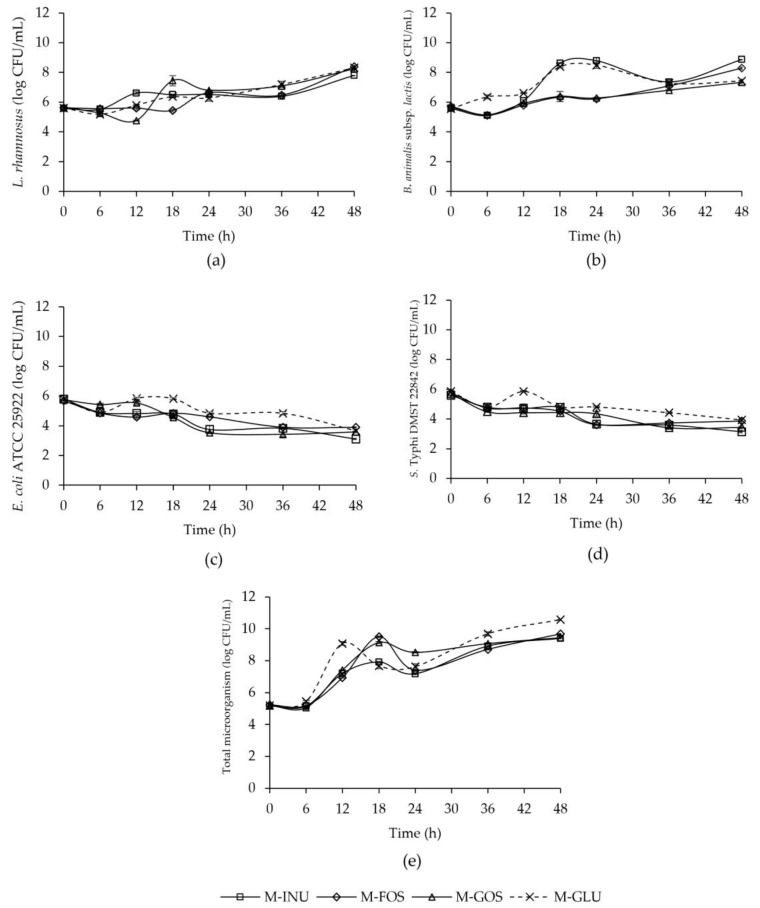
The growth kinetics of (**a**) *L. rhamnosus*, (**b**) *B. animalis* subsp. *lactis*, (**c**) *E. coli* ATCC 25922, (**d**) *S.* Typhi DMST 22842, and (**e**) total bacteria for co-culture bacterial strains in the different mediums supplemented with glucose (control) and prebiotics.

The numbers of probiotics, *L. rhamnosus* (Figure 3a) and *B. animalis* subsp. *lactis* (Figure 3b), as well as total bacteria (Figure 3e) in M-GLU co-culture, showed an upward kinetic trend ranging from 5.17 ± 0.13 to 8.32 ± 0.02, 5.56 ± 0.12 to 8.49 ± 0.07, and 5.19 ± 0.02 to 10.57 ± 0.04 log CFU/mL, respectively. However, in the case of pathogens (*E. coli* and *S*. Typhi), the kinetic trend of co-culture bacteria in M-GLU decreased. The starting counts of *E. coli* (5.76 ± 0.10 log CFU/mL) and *S*. Typhi (5.87 ± 0.04 log CFU/mL) gradually decreased to 3.67 ± 0.12 and 3.93 ± 0.06 log CFU/mL at the end of the incubation period (48 h).

The growth trend of probiotics increased in the medium supplemented with all three prebiotics, with the highest counts at 48 h of incubation. The M-FOS showed the highest stimulation of the growth number of *L. rhamnosus*, from 5.63 ± 0.15 to 8.39 ± 0.07 log CFU/mL, while the M-GOS and M-INU stimulated growth numbers, from 5.66 ± 0.02 and 5.58 ± 0.13 to 8.23 ± 0.10 and 7.79 ± 0.02 log CFU/mL, respectively, as shown in Figure 3a. The M-INU showed the highest count of *B. animalis* subsp. *lactis* from 5.58 ± 0.12 to 8.87 ± 0.12 log CFU/mL, while the M-FOS and M-GOS stimulated growth numbers from 5.71 ± 0.04 and 5.67 ± 0.07 to 8.28 ± 0.05 and 7.33 ± 0.09 log CFU/mL, respectively (Figure 3b). Similarly, the number of total bacteria had increased in M-INU, MFOS, and M-GOS (Figure 3c) and had reached its highest level at 48 h of incubation: 9.40 ± 0.03; 9.67 ± 0.02; and 9.45 ± 0.08 log CFU/mL, respectively.

In contrast, the growth number in the medium supplemented with all three prebiotics showed a declining tendency during incubation time in the case of the pathogenic number in co-culture, as shown in Figure 3c,d. Over the duration of 48 h of incubation, the amount of *E. coli* in a M-INU, M-FOS, and M-GOS dropped from 5.80 ± 0.11, 5.68 ± 0.22, 5.76 ± 0.19 log CFU/mL to 3.11 ± 0.03, 3.90 ± 0.02, and 3.58 ± 0.06 log CFU/mL, respectively. *S.* Typhi counts in M-INU, M-FOS, and M-GOS decreased from 5.60 ± 0.23, 5.76 ± 0.08, and 5.72 ± 0.07 log CFU/mL to 3.15 ± 0.08, 3.86 ± 0.10, and 3.44 ± 0.08 log CFU/mL, respectively.

### 3.4. Prebiotic Index (PI) Score of Co-Culture Bacteria on Different Medium Supplemented Prebiotics

**Figure 4:**
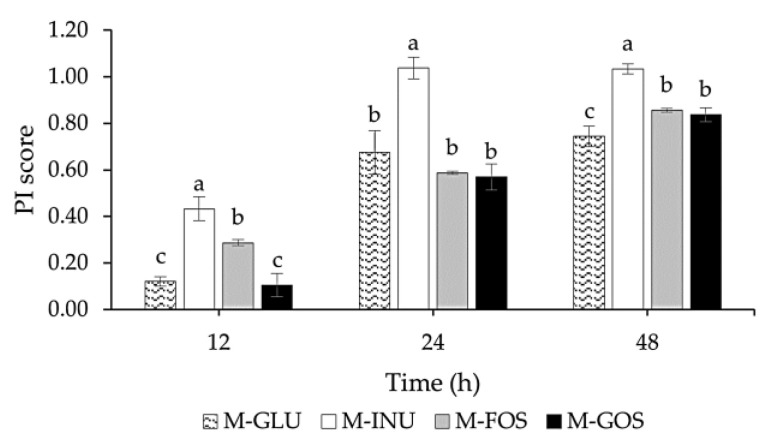
The PI score of co-culture bacterial strains in the different mediums supplemented with glucose (control) and prebiotics at 12, 24, and 48 h of incubation time, for which the various letters showed a significant mean difference (*p* < 0.05) in each incubation time.

Figure 4 shows PI scores of co-culture bacteria in various culture media at 12, 24, and 48 h of incubation time. In this study, the culture medium with glucose (M-GLU) was used as a control. At 12 h of incubation, the PI scores of M-INU (0.43 ± 0.05) and M-FOS (0.29 ± 0.01) were significantly higher than M-GLU (0.12 ± 0.02), while the PI score of M-GOS (0.10 ± 0.05) showed no significant difference compared with the control. No statistically significant difference was found in the PI score between M-FOS (0.59 ± 0.01), M-GOS (0.57 ± 0.06), and M-GLU (0.68 ± 0.09) at 24 h of incubation. However, at 48 h of incubation, the PI score for M-FOS (0.86 ± 0.01) and M-GOS (0.84 ± 0.03) significantly differed from that of the control group (0.74 ± 0.04). Interestingly, M-INU exhibited obviously the highest PI score throughout the incubation period, with M-INU values at 24 h (1.04 ± 0.05) and 48 h (1.03 ± 0.02) expressing more than 1.

### 3.5. Organic Contents of Co-Cultures in Different Medium Supplemented Prebiotics

**Figure 5:**
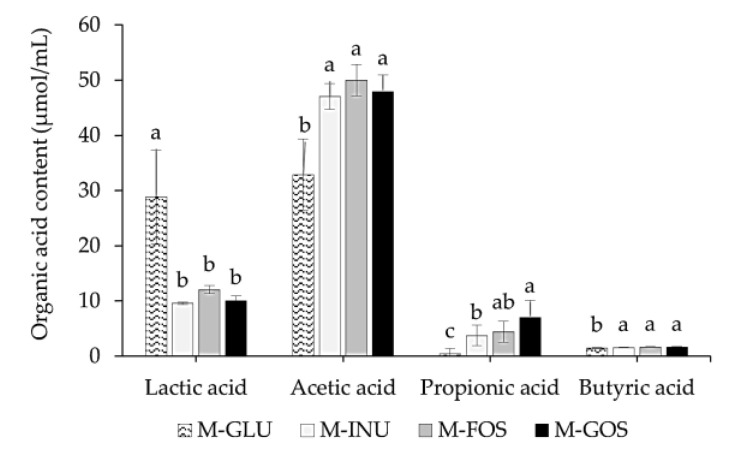
The organic acid concentration: lactic acid, acetic acid, propionic acid, and butyric acid, of co-culture bacterial strains in the different mediums supplemented with glucose (control) and prebiotics, for which the various letters showed a significant mean difference (*p* < 0.05) in each type of organic acid.

In chromatography, the retention times of lactic acid, acetic acid, propionic acid, and butyric acid were 15.64, 17.72, 19.92, and 23.03 min, respectively. A coefficient of determination (R^2^) was 0.999 of lactic acid, 0.999 of acetic acid, 0.999 of propionic acid, and 0.999 of butyric acid, which were acceptable requirements (0.995 to 1.000) [31]. The results measured the presence of organic acids in different culture media at 48 h of incubation time using HPLC, as shown in Figure 5. Lactic acid content in M-GLU (28.86 ± 8.54 µmol/mL) was significantly higher than that in M-INU, M-FOS, and M-GOS: 9.59 ± 0.23, 12.04 ± 0.74, and 10.08 ± 0.83 µmol/mL, respectively. On the other hand, M-GLU had significantly lower levels of acetic acid, propionic acid, and butyric acid than other media. Acetic acid amounts in M-INU (47.09 ± 2.34 µmol/mL), M-FOS (50.00 ± 2.82 µmol/mL), and M-GOS (47.89 ± 3.07 µmol/mL) were all significantly higher (*p* < 0.05) than in M-GLU (32.90 ± 6.45 µmol/mL). Furthermore, the propionic acid concentrations of M-INU (3.75 ± 1.85 µmol/mL), M-FOS (4.43 ± 1.92 µmol/mL), and M-GOS (7.00 ± 3.10 µmol/mL) were significantly greater (*p* < 0.05) than that of M-GLU (0.42 ± 0.98 µmol/mL). Butyric acid concentrations in M-INU, M-FOS, and M-GOS were 1.58 ± 0.04, 1.66 ± 0.05, and 1.65 ± 0.05 µmol/mL, respectively, and were significantly differently (*p* < 0.05) greater than those in M-GLU (1.48 ± 0.08 µmol/mL).

### 3.6. Optimization of Prebiotic Ratio in Culture Medium

The combination of prebiotics to achieve high quality was optimized using RSM, using the PI score and total SCFA contents as responses. Table 2 lists the actual and predicted outcomes of 17 experimental runs according to the BBD. The actual results varied between 0.05 and 1.03 for the PI score and 49.89 and 85.55 µmol/mL for the total SCFA content. The predicted data ranged from 0.02 to 0.95 in the PI score and 48.50 to 85.15 µmol/mL in the total SCFA content. Table 3 and Table 4 display the results of an analysis of variance (ANOVA) using a 2FI (interaction between two factors) model for the PI score and a quadratic model for the total SCFA contents. The outliers were excluded from the data analysis. The response models were found to be extremely significant, with *p*-values less than 0.0001. The statistical significance properties of the model terms were evaluated using respective *p*-values (*p* < 0.05). The *p*-value (0.5666 of the PI score and 0.8460 of the total SCFA contents) for “lack of fit” was insignificant relative to the error. According to the fit statistics of the PI score, the determined coefficient (R^2^), adjusted R^2^, and predicted R^2^ were, respectively, 0.9952, 0.9903, and 0.9638, while R^2^, adjusted R^2^, and predicted R^2^ of the total SCFA contents were 0.9763, 0.9458, and 0.9057, respectively (Table 5). These data indicated that the model equations were adequate for predicting responses under a combination of variable factors. The regression equation of the predicted responses of the PI score and total SCFA contents was expressed in the 2FI equation and quadratic equation as shown below.

Y_1_ = 0.4478 − 0.0017A + 0.0317B + 0.1100C − 0.1100AB − 0.1628AC − 0.2628BC (3)

Y_2_ = 53.95 − 3.59A + 0.8657B − 2.34C − 1.92AB − 7.77AC − 4.73BC + 4.12A^2^ − 4.92B^2^ + 18.08C^2^ (4)

where Y_1_ is the PI score, Y_2_ is the total SCFAs concentration (µmol/mL), and A, B, and C are the code factors of −1, 0, and 1, respectively.

**Figure 6:**
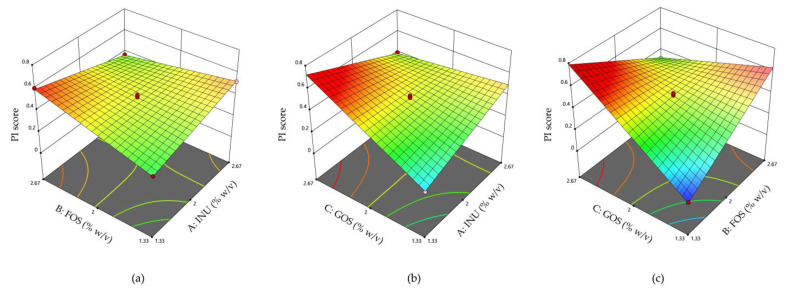
Response surface plots of the PI score for the interactions between the independent factors: (**a**) effect of INU and FOS; (**b**) effect of INU and GOS; and (**c**) effect of FOS and GOS.

**Figure 7:**
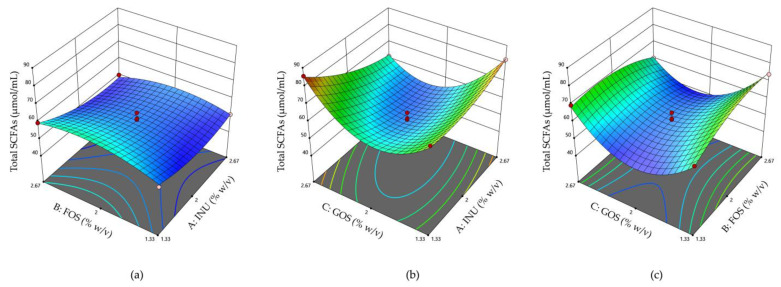
Response surface plots of the total SCFAs for the interactions between the independent factors: (**a**) effect of INU and FOS; (**b**) effect of INU and GOS; and (**c**) effect of FOS and GOS.

The interactions of all factors and their effects on the PI score and SCFA content were shown as a response surface plot, with red representing the greatest value and blue representing the minimum value (Figure 6 and Figure 7). The run number 16, with 1.33% *w/v* of inulin, 2.00% *w/v* of FOS, and 2.67% *w/v* of GOS, showed the maximum growth stimulation of probiotics with 1.03 ± 0.07 of the PI scores, and had the highest total SCFA content (85.55 ± 12.49 µmol/mL).

### 3.7. Phytochemical Structure Using FTIR

**Figure 8:**
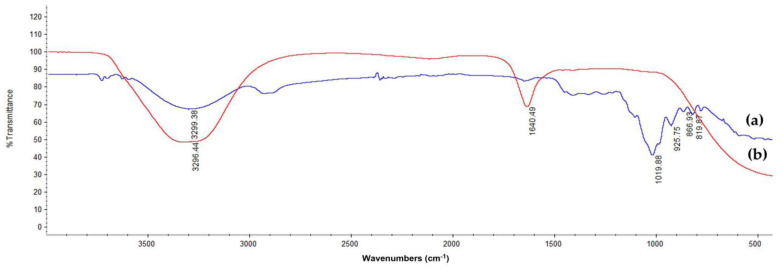
The FTIR spectrum of mixture prebiotics: (**a**) before being incubated with probiotics; (**b**) after being incubated with probiotics.

The FTIR pattern of the culture medium with prebiotics (according to the ratio at run number 16) was compared between before and after probiotic culturing, as shown in Figure 8. The FTIR spectra of the culture medium with prebiotics before incubation showed high bands at 1000 to 800 cm^−1^ and broad bands between 3600 and 3000 cm^−1^, while after incubation the high bands were at 1640 cm^−1^ and the broad bands were noticed between 3600 and 3000 cm^−1^.

## 4. Discussion

More researchers in recent years have been combining methods from several fields in an effort to understand the complex interactions that form between dietary components and health impacts. Currently, many techniques are being applied to the research of prebiotics, probiotics, and synbiotics to resolve issues related to safety, quality, function, and nutrition [32]. Because the efficiency of prebiotics might be unique to some probiotic species and strains, different probiotic species and strains exhibit totally distinct impacts, making it difficult to investigate a specific product. In this study, the optimization of the combined INU, FOS, and GOS as prebiotics to stimulate the growth of the represented probiotics and their functions were examined. The different culture media supplemented with various prebiotics (M-INU, M-FOS, and M-GOS) were investigated.

*L. rhamnosus* and *B. animalis* subsp. *lactis*, which are mainly abundant in the small intestine and colon, respectively, were employed as representative probiotic strains. According to a statement from the Thai Ministry of Public Health, these strains were included on the list of probiotics approved for use in foodstuffs. Moreover, *L. rhamnosus* appears to be the main homofermentative *Lactobacillus* species inhabiting the human gastrointestinal system. Many clinical studies have focused on selected strains: the effect of *L. rhamnosus* GG on energy metabolism and gut microbiota in obese mice [33] and the effect of *B. animalis* subsp. *lactis* BB-12 on improving the human gut microbiota [34]. In the case of monoculture (Figure 1), all culture mediums could promote the growth of *L. rhamnosus* and *B. animalis* subsp. *lactis*, but not of both pathogenic bacteria, *E. coli* and *S*. Typhi. In the same way, Figueroa-Gonzalez et al. [15] studied the ability of five probiotics (including *L. casei* Shirota, *L. casei* 1, *L. casei* 2, *L. rhamnosus* GG, and *L. rhamnosus*) to estimate the growth of behavior in three different prebiotics (INU, GOS, and lactulose). According to the result, all tested probiotics were capable of growing on medium supplemented with the studied prebiotics; however, the growth of the selected probiotics at each incubation time occurred in a different manner. Interestingly, all probiotics, except *L. casei* Shirota, in INU and GOS had a higher final growth rate and final growth than that of the control (lactose). Related studies reported that the specification of the substrate and enzyme affected the growth rate. It might be that lactobacilli in the fermentation process produced a specific enzyme to digest the prebiotics as a carbohydrate substance, resulting in carbohydrate catabolism. Glycolysis is the main pathway, converting glucose to pyruvate while producing an amount of ATP. Depending on the microorganism involved, pyruvate is transformed into several end products, such as lactic acid, ethanol, or other organic substances. Fermentation is an inefficient method of producing energy; nevertheless, it allows microorganisms to grow, resulting in growth benefits [35,36]. Moreover, the structures influence carbohydrate digestion, and fermentability is one of the physicochemical characteristics of the various fibers. Because they regulate the exposed surface area to bacterial degradation, fiber particle size and degree of solubility have a significant impact on the susceptibility of fibers to bacterial fermentation [37].

This research determined the specific growth rate because it represented an increase in the population during a certain time period. *L. rhamnosus* exhibited the maximum specific growth rate in M-GOS (*p* < 0.05), while *B. animalis* subsp. *lactis* showed the highest specific growth rate in M-FOS (*p* < 0.05) (Figure 2). Similarly, *L. reuteri* C1 and C6 demonstrated the best growth concerning basal MRS media containing GOS (*p* < 0.05) when compared with other carbon sources [23]. Some factors allowing lactic acid bacteria (LAB) to reach their maximal specific growth rate in GOS include their enzyme mechanism. One related enzyme allowing LAB to break down and utilize GOS is β-galactosidase, which is a common enzyme in many microorganisms, including *Lactobacillus* species [38]. This process of breaking the β-glycosidic link between galactose molecules in GOS releases free galactose that *L. rhamnosus* can utilize as a source of carbon and energy. In another study, among the tested carbohydrate sources, FOS was the most effective in enhancing the growth rate of *Bifidobacterium* Bf-1 and Bf-6 in skim milk [39]. Most bifidobacteria degrade and use FOS because they contain a competitive fructofuranosidase enzyme, which is abundantly produced by bifidobacteria in culture [38].

The diversity and interactions observed in their natural surroundings might not be fully represented in a monoculture, referring to a single-species culture of microorganisms. On the other hand, a co-culture, a culture containing multiple species of microorganisms, might more effectively represent the complexity of actual microbial communities. Under co-culture conditions, the culture medium with prebiotics could also stimulate the growth of both probiotics but not affect the growth of the two pathogenic bacteria, *E. coli* and *S*. Typhi (Figure 3). Buddington et al. [40] demonstrated that INU and FOS offered effective protection against the pathogens *S*. Typhimurium and *Listeria monocytogenes* in mice with abnormal crypt foci in the colon and in a cell line. Moreover, the *Bifidobacterium*, together with prebiotic transgalactosylated oligosaccharides (TOS), could be used for the anti-infective activity against *Salmonella* in a murine model [41].

To evaluate the prebiotic potential of different foods and ingredients, PI was employed as a measure to assist in selecting prebiotic-rich foods. PI value was calculated by comparing the increase in the growth of probiotic bacteria (if an increase in the populations is a positive effect) in the presence of the ingredient to the growth of less desirable ones (if an increase in the populations is a negative effect) [15,42,43]. Figure 4 displays a comparison of PI values over the same time period for various prebiotics. M-INU showed the highest significant difference (*p* < 0.05) from others. In addition, the PI score of all culture mediums containing prebiotics (M-INU, M-FOS, and M-GOS) showed significantly different (*p* < 0.05) values than those of the control (M-GLU) at 48 h of incubation. Interestingly, the PI scores of M-INU at 24 h and 48 h of incubation had values higher than 1. The fact that the ratio is greater than 1 shows that the growth stimulation of the studied probiotic bacteria, such as *L. rhamnosus* and *B. animalis* subsp. *lactis*, was higher than the growth stimulation of pathogenic bacteria, such as *E. coli* -and *S*. Typhi. In other words, M-INU had a positive effect on the growth of beneficial bacteria and a negative effect on the growth of potentially harmful bacteria. Similarly, Ghoddusi et al. [18] reported that INU, FOS, polydextrose, and isomaltooligosaccharides, both individually and in combination, had an impact on the PI value. According to the study, INU had a positive effect on the growth of beneficial bacteria at 24 h but a negative impact at 8 h. In another report, PI scores of 0.91 for INU, 0.56 for FOS, and 5.19 for GOS were found in a prior study that was conducted at pH 6 with a concentration of 2% *w/v* [26]. Prebiotics and mixed prebiotics have different chemical structures and functionalities that influence their fermentation by gut bacteria, leading to an impact on the PI. Some prebiotics, e.g., FOS, GOS, and XOS, are more quickly fermented by beneficial gut bacteria, resulting in a high prebiotic index score. Moreover, the degree of polymerization (DP) or the number of sugar units in a prebiotic molecule also affects its fermentability and prebiotic index score. Prebiotics with a low DP, such as FOS (DP of 2 to 8), GOS (DP of 2 to 8), and XOS (DP of 2 to 10), tend to be more fermentable and have a higher prebiotic index score than prebiotics with a high DP, such as INU (DP of 2 to 60) [44,45,46]. This is because the longer one contains fewer non-reducing ends per unit mass than the shorter one, resulting in less substrate for hydrolysis by bacterial enzymes. However, resistant starch and polydextrose are examples of prebiotics that may need prolonged fermentation periods or may require other bacterial species in order to reach the same prebiotic index score as other prebiotics [46]. Studies have reported that many genes supported healthy digestion by regulating prebiotic metabolism and the immune response to probiotics [15]. For example, genes encoding enzymes such as β-galactosidase and β-fructofuranosidase are involved in the breakdown of prebiotic GOS and FOS, respectively [47,48]. These factors could alter different PI values. Notably, the PI score value was simply one of several methods used to determine the prebiotic ability, and was not a guarantee that a product or diet was safe and efficient as a prebiotic. Therefore, it becomes necessary to consider other beneficial substances, such as organic acid.

Prebiotics are indigestible food ingredients that typically pass through the gastrointestinal tract and are fermented by gut bacteria, leading to the main production of SCFAs such as acetic acid, propionic acid, and butyric acid, as well as other beneficial substances such as lactic acid [49]. These SCFAs play important roles in maintaining gut balance and influencing the metabolic system. Additionally, lactic acid can assist in lowering the pH of the gastrointestinal tract and can create a more suitable environment for good bacteria. In the present study, the concentration of lactic acid in M-GLU was significantly higher (*p* < 0.05) than in all culture media with prebiotics (M-INU, M-FOS, and M-GOS). In contrast, the SCFAs concentration of all media containing prebiotics was significantly greater (*p* < 0.05) than that of M-GLU. It might be that GLU is a readily available source of energy and carbon that produces lactic acid through glycolysis. It constitutes a rapid and efficient way for microorganisms to produce the substance under anaerobic conditions. However, the production of SCFAs through the fermentation of prebiotics requires a different metabolic pathway that is typically less efficient than glycolysis. This can contribute to the relative abundance of lactic acid compared to SCFAs in a culture medium containing GLU. Furthermore, no significant difference (*p* > 0.05) was found in the organic acid concentrations of any prebiotic. The quantity of the organic acid could be in the following order: acetic acid > propionic acid > butyric acid, as shown in Figure 5. Similarly, a related study revealed that the total SCFAs concentration had a slight increase in the three-stage continuous culture system after treating with durum wheat dietary fiber (DWF) and enzyme-treated DWF. However, no differences were found between the two tested DWF regarding the percentage of SCFAs in their composition [50]. In fact, the SCFAs concentration was found in the following order: acetic acid > propionic acid > butyric acid [50]. Similarly, the SCFAs production in batch culture by G12, G19, G37-glucooligosaccharide, G12, G19, G37-maltodextrin, and INU were related to concentration in the order acetic acid > propionic acid > butyric acid [20]. SCFAs production in co-culture conditions might interrupt the growth of selected pathogenic bacteria. This is supported by the fact that *Bifidobacterium* or *Lactobacillus* generate SCFAs or other substances inhibiting pathogens and resulting in reduced intestinal pH [9]. These suggest that SCFAs may be a critical mechanism by which prebiotics promote health.

Overall, learning more about mixed prebiotics is important for many reasons. These include (i) advancing our knowledge of gut health: different prebiotics interact with one another and the gut microbiome, providing a better understanding of gut health and information about improving it; (ii) creating functional foods: combined prebiotics may be more helpful in terms of gut health and overall wellness; (iii) developing individual nutrition recommendations: this can assist individuals in gaining the maximum health advantages from the food by selecting the prebiotics that are most beneficial for their specific gut microbiome; and (iv) treating gut illnesses: researchers can develop more effective treatments for these conditions, such as irritable bowel syndrome (IBS) and inflammatory bowel disease (IBD). Prebiotics, either alone, in mixtures, or combined with probiotics in the form of synbiotics, can improve the gastrointestinal health of humans [51] Therefore, the present study optimized the ratio of different prebiotics, INU, FOS, and GOS, based on a related study in 4 to 8 g/day of the total formulation [21], by using BBD of response surface methodology with 3 levels and 3 factors (Table 1). The response surface method has become one of the most used optimization approaches to create the best conditions with a minimum number of experiments [52]. In addition, BBD has been accepted as a good design for the optimization of the main variables. The result in this study found that run 16 with the ratio 1.33% *w*/*v* of inulin, 2.00% *w*/*v* of FOS, and 2.67% *w*/*v* of GOS, in the presence of a higher concentration of GOS, had significantly different PI scores (1.03 ± 0.007) and total SCFA concentration (85.55 ± 12.49 µmol/mL) (Table 2). This is confirmed by the analysis of variance for the 2FI model of the PI score, which is significant (*p* < 0.0001) for the model and not significant (*p* = 0.5666) for the lack of fit (Table 3). In addition, the total SCFA concentrations were confirmed by the quadratic model, which was significant (*p* < 0.0001) for the model and not significant (*p* = 0.8460) for the lack of fit (Table 4). These indicate that the generated Equations (3) and (4) could be used to predict the optimal component for the PI score and total SCFA production. Thus, run 16 is an appropriate ratio of tested prebiotics for further study. The 3D response surface expressed the interaction between the effects of INU-GOS, and FOS-GOS on both responses, with the PI score and total SCFA production higher than the interaction of INU and FOS (Figure 6 and Figure 7).

Similarly, the optimized combination of prebiotics in the related study was 1.26% *w*/*v* of FOS, 6.75% *w*/*v* of GOS, and 0.99% *w*/*v* of INU, where the higher concentration of GOS in the prebiotic mix was seen. Many reports have been conducted regarding the effectiveness of the mixed prebiotics. GOS as a prebiotic was proven to have an advantage over other substrates [51]. In another study, the amount of SCFA production was obtained as a total of acetic, butyric, and propionic acids. Only the probiotic produced significant quadratic effects on SCFA production, as did interactions between probiotics and FOS, and probiotics and maltodextrin. The response surface indicated that the production of SCFA from the fermentation of FOS was closely associated with the uptake of the substrate [53]. The ability of the lactobacilli and bifidobacteria to ferment specific oligosaccharides and polysaccharides can be important in the development of synbiotics [51].

The FTIR spectra were used to check the purity, identify a biomolecule, and indicate the presence of a functional group. The high-spectra band of mixture prebiotics before incubation was between 1000 and 800 cm^−1^, which is the range of carbohydrates or oligo- and polysaccharides. The IR spectra of carbohydrates can be divided into three specific spectral regions, including 1200 to 900 cm^−1^, 3000 to 2700 cm^−1^, and 900 to 600 cm^−1^ [54]. The spectral region between 1200 and 900 cm^−1^ is generally dominated by a complex sequence of intense peaks due mainly to strongly coupled C-C, C-O stretching, and C-O-H, C-O-C deformation modes of various oligo- and polysaccharides [54]. The FTIR spectra showed an increase over time in the polysaccharide-oligosaccharide region (1200 to 900 cm^−1^) for the conditions tested [55] In this study, the spectra band of the mixed prebiotics after incubation did not show a peak between 1000 and 800 cm^−1^; it may be that the structure of the polysaccharide was digested by the fermentation of probiotics (Figure 8). The spectra band of the mixture of prebiotics after incubation was 1640 cm^−1^. The previous studies reported the same result: the FTIR major peak after the fermentation process of yogurt was observed at 1640 cm^−1^ [56].

The different prebiotics can stimulate the growth of different probiotics and enhance their function. Inulin, one of the prebiotics, was commonly used as an ingredient in the functional food industry. However, purity INU, a long chain polysaccharide, tends to be costly and is extracted from chicory root, artichoke, and asparagus. On the other hand, FOS and GOS are short chain oligosaccharides that are derived from sources such as sugar (sugar found in milk for GOS) or cane. However, different sources may have slightly different properties, and probiotic specifications. Consequently, it is important to note that the optimization of a mixed prebiotic ratio may result in a potential functional food ingredient. Additionally, this knowledge of certain components influences the beneficial development of a dosage for our further clinical trials.

## 5. Conclusions

In this study, all prebiotics (INU, FOS, and GOS) had the potential to stimulate the growth of probiotics, *L. rhamnosus* and *B. animalis* subsp. *lactis*, and showed a high prebiotic index and SCFA concentrations when compared with of the control, but did not affect the growth of *E. coli* and *S*. Typhi. The optimal ratios of the three different prebiotics having a significant impact on the prebiotic index and SCFA production were 1.33% *w*/*v* of INU, 2.00% *w*/*v* of FOS, and 2.67% *w*/*v* of GOS (Run 16). In the future, suitable ratios of mixed prebiotics will be used as synbiotics for alternative food supplements in order to improve probiotic stimulation, or balance the gut microbiota in clinical trials with human volunteers.

## Figures and Tables

**Table 1:** The content and code of an independent variable on a Box–Behnken design (BBD) for PI score and total short chain fatty acids (SCFAs).

Std	Run	Variable Factors (%*w*/*v*)		
		X1: INU	X2: FOS	X3: GOS
9	1	2.00 (0)	1.33 (−1)	1.33 (−1)
14	2	2.00 (0)	2.00 (0)	2.00 (0)
4	3	2.67 (1)	2.67 (1)	2.00 (0)
15	4	2.00 (0)	2.00 (0)	2.00 (0)
3	5	1.33 (−1)	2.67 (1)	2.00 (0)
6	6	2.67 (1)	2.00 (0)	1.33 (−1)
13	7	2.00 (0)	2.00 (0)	2.00 (0)
5	8	1.33 (−1)	2.00 (0)	1.33 (−1)
8	9	2.67 (1)	2.00 (0)	2.67 (1)
12	10	2.00 (0)	2.67 (1)	2.67 (1)
10	11	2.00 (0)	2.67 (1)	1.33 (−1)
11	12	2.00 (0)	1.33 (−1)	2.67 (1)
2	13	2.67 (1)	1.33 (−1)	2.00 (0)
1	14	1.33 (−1)	1.33 (−1)	2.00 (0)
16	15	2.00 (0)	2.00 (0)	2.00 (0)
7	16	1.33 (−1)	2.00 (0)	2.67 (1)
17	17	2.00 (0)	2.00 (0)	2.00 (0)

**Table 2:** The actual and predicted values of the PI score and total SCFAs for the mixed prebiotics following the run of a Box–Behnken design (BBD).

Std	Run	Variable Factors (%*w*/*v*)			PI Score ^1^		Total SCFAs ^1^	
		X1: INU (%*w*/*v*)	X2: FOS (%*w*/*v*)	X3: GOS (%*w*/*v*)	Actual Value	Predicted Value	Actual Value	Predicted Value
9	1	2.00 (0)	1.33 (−1)	1.33 (−1)	0.05 ^f^	0.02	64.33 ^bcde^	63.85
14	2	2.00 (0)	2.00 (0)	2.00 (0)	0.44 ^bcd^	0.45	55.00 ^de^	53.95
4	3	2.67 (1)	2.67 (1)	2.00 (0)	0.37 ^cd^	0.38	49.89 ^d^	48.50
15	4	2.00 (0)	2.00 (0)	2.00 (0)	0.47 ^bcd^	0.45	58.52 ^cde^	53.95
3	5	1.33 (−1)	2.67 (1)	2.00 (0)	0.60 ^bc^	0.65	59.60 ^cde^	59.52
6	6	2.67 (1)	2.00 (0)	1.33 (−1)	0.61 ^b^	0.69	82.26 ^ab^	82.66
13	7	2.00 (0)	2.00 (0)	2.00 (0)	0.45 ^bcd^	0.45	54.42 ^de^	53.95
5	8	1.33 (−1)	2.00 (0)	1.33 (−1)	0.17 ^ef^	0.21	75.21 ^abc^	74.30
8	9	2.67 (1)	2.00 (0)	2.67 (1)	0.40 ^bcd^	0.36	61.54 ^cde^	62.45
12	10	2.00 (0)	2.67 (1)	2.67 (1)	0.32 ^de^	0.35	60.42 ^cde^	60.90
10	11	2.00 (0)	2.67 (1)	1.33 (−1)	0.46 ^bcd^	0.38	74.05 ^abcd^	75.04
11	12	2.00 (0)	1.33 (−1)	2.67 (1)	0.38 ^cde^	0.46	69.62 ^abcde^	68.63
2	13	2.67 (1)	1.33 (−1)	2.00 (0)	0.52 ^bcd^	0.47	50.54 ^d^	50.62
1	14	1.33 (−1)	1.33 (−1)	2.00 (0)	0.31 ^de^	0.31	52.55 ^d^	53.94
16	15	2.00 (0)	2.00 (0)	2.00 (0)	0.44 ^bcd^	0.45	51.30 ^d^	53.95
7	16	1.33 (−1)	2.00 (0)	2.67 (1)	1.03 ^a^	0.95	85.55 ^a^	85.15
17	17	2.00 (0)	2.00 (0)	2.00 (0)	0.43 ^bcd^	0.45	50.49 ^d^	53.95

^1^ PI score and total SCFAs concentration (µmol/mL) were determined at 48 h of incubation in duplicate. The different superscript letters indicated a significant difference (*p* < 0.05) between the same columns of experimental runs.

**Table 3:** Analysis of variance (ANOVA) for the 2FI model of the PI score with a Box–Behnken design (BBD) experiment.

Source	Sum of Squares	df	Mean Square	F-Value	*p*-Value	
Model	0.2514	6	0.0419	205.70	<0.0001	significant
A	0.0000	1	0.0000	0.0655	0.8066	
B	0.0048	1	0.0048	23.63	0.0028	
C	0.0323	1	0.0323	158.40	<0.0001	
AB	0.0484	1	0.0484	237.60	<0.0001	
AC	0.0434	1	0.0434	212.85	<0.0001	
BC	0.1130	1	0.1130	554.70	<0.0001	
Residual	0.0012	6	0.0002			
Lack of fit	0.0003	2	0.0002	0.6570	0.5666	not significant
Pure error	0.0009	4	0.0002			
Cor total	0.2526	12				

A, B, C, AB, AC, and BC represent INU, FOS, GOS, interaction between INU and FOS, interaction between INU and GOS, and interaction between FOS and GOS, respectively.

**Table 4:** Analysis of variance (ANOVA) for the quadratic model of the total SCFAs with a Box–Behnken design (BBD) experiment.

Source	Sum of Squares	df	Mean Square	F-Value	*p*-Value	
Model	2039.98	9	226.66	32.03	<0.0001	significant
A	102.87	1	102.87	14.54	0.0066	
B	6.00	1	6.00	0.8474	0.3879	
C	43.87	1	43.87	6.20	0.0416	
AB	14.81	1	14.81	2.09	0.1913	
AC	241.19	1	241.19	34.09	0.0006	
BC	89.48	1	89.48	12.65	0.0093	
A^2^	71.38	1	71.38	10.09	0.0156	
B^2^	101.87	1	101.87	14.40	0.0068	
C^2^	1375.74	1	1375.74	194.44	<0.0001	
Residual	49.53	7	7.08			
Lack of fit	8.29	3	2.76	0.2680	0.8460	not significant
Pure error	41.24	4	10.31			
Cor total	2089.51	16				

A, B, C, AB, AC, BC, A^2^, B^2^, and C^2^ represent INU, FOS, GOS, interaction between INU and FOS, interaction between INU and GOS, interaction between FOS and GOS, quadratic term of INU, quadratic term of FOS, and quadratic term of GOS, respectively.

**Table 5:** Fit statistics of the PI score and total SCFAs with a Box–Behnken design (BBD) experiment.

Statistical Parameter	PI Score	Total SCFAS
Standard deviation	0.0143	2.66
Mean	0.3823	62.08
Coefficient of variation (% CV)	3.73	4.29
Coefficient of determination (R^2^)	0.9952	0.9763
Adjust R^2^	0.9903	0.9458
Predicted R^2^	0.9638	0.9057
Adequate precision	52.3031	17.9638

## Data Availability

The data presented in the manuscript are available on request from the corresponding author.
